# The Impact of Melatonin on Cellular Dynamics and Gene Expression of Bovine Embryos Cultured Under Low and High Oxygen Tension

**DOI:** 10.1002/mrd.70102

**Published:** 2026-04-06

**Authors:** Isabella Rodrigues dos Santos Oliveira, Carlos Frederico Martins, Fabiana Lima Rodrigues, Victor Carlos Mello, Maria Tereza de Oliveira Rodrigues, Lucas Costa de Faria, Hallya Beatriz Sousa Amaral, Rosângela Vieira de Andrade, Marcio José Poças Fonseca, Margot Alves Nunes Dode, Sônia Nair Báo

**Affiliations:** ^1^ Departamento Biologia Celular Universidade de Brasília Brasília Brasil; ^2^ Embrapa Cerrados Brasília Brasil; ^3^ ICESP Brasília Brasil; ^4^ Departamento Saúde Animal Universidade de Brasília Brasília Brasil; ^5^ Programa de Pós‐graduação em Ciências Genômicas e Biotecnologia Universidade Católica de Brasília Brasília Brasil; ^6^ Embrapa Recursos Genéticos e Biotecnologia Brasília DF Brasil

**Keywords:** apoptosis, bovine embryos, gene expression, melatonin, oxidative stress

## Abstract

This study investigated the effects of melatonin supplementation in the culture medium on the development, cellular dynamics and gene expression of bovine embryos produced *in vitro* under low (5%) or high (20%) oxygen tension. Zygotes were cultured without melatonin or with (10^−9^ M), and cleavage, blastocyst rates, blastomere number, apoptosis, mitochondrial activity, lipid accumulation, and expression of genes related to metabolism, oxidative stress and embryo quality were evaluated. Under high O₂ tension, melatonin increased blastocyst rate (39.8% vs. 34.0%), raised blastomere number, reduced apoptosis, and decreased lipid accumulation. It also upregulated SOD2, KRT8, IFN‐τ, and PLAC8. Under low O₂ tension, melatonin increased cleavage rate and mitochondrial activity but did not affect blastocyst rate; only SOD2 was upregulated. Embryos cultured without melatonin under low O₂ showed higher IFN‐τ and PLAC8 expression. In conclusion, melatonin improves embryo quality and viability mainly under high oxygen tension, acting as an antioxidant, gene modulator and apoptosis inhibitor. Its effects are more limited under low O_2_, likely because this environment is closer to physiological conditions and requires fewer responses to oxidative stress.

## 1 Introduction

In vitro embryo production (IVEP) is currently an important procedure both in human clinics, to overcome reproductive problems, and in animal production, particularly for bovine multiplication. The use of *in vitro*‐produced embryos in livestock has been highly promising in recent years, following advances brought by the pharmacological control of the estrous cycle and ovulation. Embryo production by in vitro fertilization (IVF) can decisively contribute to greater reproductive efficiency. This technique stands out as a tool for the rapid multiplication of genetically superior animals, shortening the generation interval and intensifying selection, promotes greater efficiency in the control of the inbreeding rate, leading to substantial gains in the genetic improvement of cattle. Furthermore, IVF can contribute to an increase in the efficiency and sustainability of milk and meat production systems (Báez et al. 2024).

Despite the advances in bovine embryo in vitro production techniques, their efficiency is still limited by various biological and technical challenges. In particular, IVEP outcomes differ depending on whether oocytes are matured *in vivo* or *in vitro*, as *in vivo* matured oocytes develop within the physiological follicular microenvironment, which provides biochemical and biophysical cues that are only partially reproduced under *in vitro* conditions. One of the main challenges is replicating the biochemical and biophysical environment of the female reproductive tract during in vitro embryo culture (Missio et al. 2022; Ofosu et al. 2023; Báez et al. 2024). During culture, embryos frequently face suboptimal conditions, such as variations in temperature, pH, gas composition, and media constitution, which can lead to metabolic alterations and compromised development (Missio et al. 2022).

It has been established that the partial tension of oxygen in the follicle, oviduct, and uterus does not exceed 8% (Harvey 2007). However, the maturation and fertilization of oocytes, and the culture of bovine embryos, are commonly conducted with 20% O_2_. This hyperoxidative environment favors the excessive production of reactive oxygen species (ROS) and can compromise the integrity of cell membranes, proteins, and DNA, reducing cleavage and blastocyst formation rates, in addition to impairing embryo quality (Keane and Ealy 2024) and cryotolerance (Valente et al. 2022). In this context, strategies that increase the antioxidant defense of embryos have been widely studied to mitigate such deleterious effects. Furthermore, embryo culture under physiological oxygen tension (5%) has also been proposed as a strategy to improve the quality of in vitro produced embryos (Báez et al. 2024).

Melatonin (N‐acetyl‐5‐methoxytryptamine) has shown beneficial action both in the direct neutralization of reactive oxygen species (ROS) and in the induction of antioxidant enzymes, such as superoxide dismutase and catalase. These properties have been drawing the attention to its application in assisted reproduction protocols, especially for the *in vitro* production of bovine embryos, where oxidative stress is one of the main limitations to embryo development (Eber et al. 2017; Shi et al. 2022).

Considering that melatonin also stimulates mitochondrial function and exerts anti‐inflammatory and anti‐apoptotic effects (Ji et al. 2023), this approach represents an alternative not only to mitigate the harmful effects of ROS during embryo culture under high O₂ tension (Zhao et al. 2019), but also to improve embryo quality in low O₂ culture systems. Aiming the combination of controlled environments and the strategic use of antioxidants, the objective of this study was to investigate the effects of melatonin supplementation on the production of bovine embryos cultured simultaneously under conditions of low and high oxygen tension, in order to evaluate if this interaction can contribute to the improvement of embryo quality and the optimization of bovine assisted reproduction protocols.

## 2 Materials and Methods

This study was carried out at the Animal Reproduction Laboratory of Embrapa Cerrados and Microscopy and Microanalysis Laboratory of University of Brasilia. It was approved by the Ethical Committee of the Embrapa Cerrados (protocol 800‐4372‐1/2020).

### 2.1 Experimental Design

The experiment evaluated the effect of melatonin (10^‐9 ^M) in the *in vitro* production of bovine embryos cultured under different oxygen tensions: high oxygen tension (5% CO_2_ and 20% O_2_) and low oxygen tension (5% CO_2_, 5% O_2_, 90% N_2_). In experiments conducted in our laboratory, this melatonin concentration led to an improvement of embryo production and viability (Marques et al. 2020).

After maturation and fertilization, the possible zygotes were randomly assigned to one of the treatments: 1) high O_2_ with melatonin (HI‐MEL+), 2) low O_2_ with melatonin (LO‐MEL+), 3) high O_2_ without melatonin (HI‐MEL−), 4) low O_2_ without melatonin (LO‐MEL−).

To evaluate the effect of treatments in the mo embryo development, cleavage rate on day 2 (D2) and blastocyst rate on day 7 of culture (D7) were monitored. In addition, in the expanded blastocysts, number of blastomeres, percentage of apoptosis, mitochondrial activity, lipid metabolism, and expression of genes related to oxidative stress and embryo quality were evaluated to identify the contribution of melatonin in the different experimental systems.

### 2.2 Oocyte Selection, Maturation, Fertilization and Embryo Culture

Bovine ovaries were collected from a local slaughterhouse, maintained in saline solution 0.9% at 36°C, transported to the laboratory, and processed within 4 h. Follicles from 3 to 8 mm diameter were aspirated using 40 × 12 needles and syringes to collect the cumulus–oocyte complexes (COCs).

For 20 replicates of IVF, groups of 30 grade I and II COCs, classified as per the method used by Papis et al. (2007) were selected and incubated in 200 μL drops of in vitro maturation (IVM) medium at 38.5°C in an atmosphere of 5% CO_2_ in air. The IVM medium contained TCM‐199 with Earle's salts and l‐glutamine (Gibco, Invitrogen Co., Grand Island, NY, USA) supplemented with 10% fetal bovine serum (v/v), 0.2 mM pyruvate, 1 mg mL^−1^ follicle stimulating hormone (Folltropin, Bioniche Co.), 75 μg mL^−1^ amikacin, and 1 mM cysteamine. After 22 h, the COCs were washed and transferred to 200 μL drops of IVF medium covered with mineral oil. The IVF medium contained TALP‐FERT (Tyrode's albumin lactate pyruvate) supplemented with 6 mg mL^−1^ fatty acid‐free bovine serum albumin, 0.2 mM pyruvate, 30 μg mL^−1^ heparin, 20 μM penicillamine, 10 μM hypotaurine, 1 μM epinephrine, and 75 μg mL^−1^ amikacin.

For in vitro fertilization, semen from a single Nellore bull of known fertility was used. After thawing (37°C for 30 s), motile spermatozoa were obtained using the Percoll column method (Komninou et al. 2016) and diluted in 100 μL Tyrode's HEPES‐buffered medium supplemented with 0.2 mM pyruvate and 75 μg mL^−1^ amikacin. The spermatozoa were then added to the fertilization drop at a final concentration of 1.0 × 10^6^ live sperm·mL^−1^. Oocytes and sperm were co‐incubated at 38.5°C in an atmosphere of 5% CO_2_ in air for 18 h, with the day of insemination considered as day 0 (D0). At 18 h after co‐incubation, the presumptive zygotes were partially denuded and cultured in four treatments: 1) Synthetic Oviduct Fluid (SOfaa) supplemented with 10^‐9 ^M melatonin in high O_2_ (20%); 2) SOfaa without melatonin in high O_2_ (20%); 3) SOfaa supplement with 10^‐9 ^M melatonin in low O_2_ (5%); 4) SOfaa without melatonin in low O_2_ (5%). The IVC medium contained SOfaa (An et al. 2019) supplemented with 2.7 mM myo‐inositol, 0.2 mM pyruvate, 2.0% fetal bovine serum (vol/vol), 5 mg mL^−1^ fatty acid‐free bovine serum albumin, and 75 μg mL^−1^ amikacin. Zygotes were then cultured until day 7 (D7) of development. Cleavage rates were assessed on day 2 (D2, 2 days post fertilization) and non‐cleaved structures were removed. On day 4 (D4) 50% of culture medium was renewed and blastocyst rates were evaluated on day 7 (D7).

### 2.3 Mitochondria and Lipids Staining

Ten expanded embryos per treatment were washed thrice in PBS supplemented with 0.3% polyvinylpyrrolidone (PVP) and incubated for 30 min with MitoTracker Deep Red FM at 400 nM (Invitrogen®, Waltham, USA), diluted in 0.9% saline solution at 36°C. For lipids staining, the methodology previously described in Faria et al. (2021) was followed. The embryos were fixed for 1 h in 4% paraformaldehyde, washed thrice in PBS, and stored at 4°C in 1% paraformaldehyde for up to 7 d. Subsequently, they were washed twice in PBS‐PVP and incubated for 30 min in PBS supplemented with 0.2% Triton X‐100 at 25°C to 27°C. After three washes in PBS‐PVP, the embryos were stained with Bodipy (Molecular Probes, Eugene, OR, USA) at a concentration of 20 µg mL^‐1^ (diluted in 50 µL of absolute ethanol and 950 µL of PBS) for 1 h. The embryos were then washed thrice in PBS‐PVP, placed on a microscope slide in a 5 μL drop of PBS with PVP, and examined under the LSM Leica SP5 microscope (Leica microsystems, New Orleans, LA, USA). All samples were analyzed and photographed with a 20× objective and a 488 nm argon laser to visualize lipid droplets, with a fluorescence spectrum between 495 and 505 nm. For mitochondrial evaluation, a 638 nm laser was used, with excitation/emission of 644/665 nm. The embryos underwent up to 20 transverse sections at 4 µm intervals. Z‐stacking was employed to create an image of overlapping sections. After constructing the final image of each embryo, they were adjusted to a grayscale (8‐bit image) using the Image J program (National Institutes of Health), and the background was corrected. Mitochondrial activity was quantified by the mean number of pixels. Lipid measurements were expressed as the ratio between the area occupied by lipids (number of pixels) and the total area of the embryo (µm). Intensity was normalized by the embryo area and an automatic threshold method was used.

### 2.4 Gene Expression Analysis in Embryo

To assess the effect of melatonin in both oxygen tension systems, the expression of genes related to embryonic quality was evaluated by quantitative reverse transcriptase real‐time PCR (qRT‐PCR). Genes related to cell metabolism (SLC2A1), oxidative stress (SOD1 and SOD2), maternal recognition (IFN‐τ), implantation (KRT8) and placentation (PLAC8) were studied. Three pools of 15 expanded blastocysts of each treatment were used.

Total RNA was isolated using the RNeasy Plus Micro Kit (Quiagen, Hilden, Germany), according to the manufacturer's instructions. The total volume of isolated RNA was used for cDNA synthesis by the First‐Strand cDNA Synthesis kit (Invitrogen, Whaltham, USA) ‐ SuperScript III (200 U/µL) and Oligo‐dT primer (0.5 µg µL^‐1^) in the final volume of 40 µL. The reactions were carried out at 65°C for 5 min and 50°C for 50 min, followed by inactivation of the enzyme at 85°C for 5 min. The qPCR analysis was performed with Fast Sybr Green Master Mix (Applied Biosystems, Forster City, USA). To promote maximum amplification efficiency for each gene (76%–110%), the reactions were optimized by calculations using the relative standard curves in the 7500 2.0.3 program (Applied Biosystems). The samples were analyzed in triplicate and the specificity of each PCR product was determined by analyzing the melting curve and the amplicon size on agarose gel. The reactions were carried out in the final volume of 25 µL using cDNA corresponding to 0.5 embryo. The conditions of the PCR cycles were 95°C for 5 min, followed by 50 cycles of denaturation at 95°C for 10 s and then annealing at 60°C for 30 s. The name, sequence and concentration of the primers, size of the amplicon and annealing temperature for each gene are listed in Table 1.

**Table 1:** Information for specific primers used to amplify gene fragments for real‐time quantitative PCR analysis.

Gene	Primer sequence	Amplicon size (bp)	Primer concentration (nM)	GeneBank Number of accession/Reference
SOD1	F‐ GGGAGATACAGTCGTGGTAA	160	300	NM_174615.2
	R: CCACAGTCAGCAATGGT GATCT			
SOD2	F: AGAAGGAGCCAGGAGGAGTG	104	300	NM_001034034.1
	R: TGGGTGATGTCAGTTGAGG			
SLC2A1	f: atccacagtgg cacagttgag	89	300	NM_174602.2
	r: ggctgagggtgg aatggtg			
KRT8	F: TGTGAAGAAGATTGAGACCCG CGA	160	300	NM_001076004.1
	R: AAACCTCAGGTCTCCTGTGC AGAT			
PLAC 8	F: GAC TGG CAG ACT GGC ATC TT	140	300	NM_016619
	R: CTC ATG GCG ACA CTT GAT CC			
IFN‐τ	F: TGCAGTACAAAGGAGAGGTGA	120	300	NM_001015511.4
	R: GAGTCTTCAAGT GGTGAAGAA			
ACTB	F: ACTCCTCCTGGTCCGAATGC	174	300	NM_001101
	R:GCCCCTAGACCAGGAAAATCC			
GAPDH	F: GTGGTCTCCTCTGACTTCAACA	150	300	NM_002406.7
	R:CTCTTCCTCTTGTGCTCTTG			

Abbreviations: ACTB, β‐actin; F, forward primer; GAPDH, glyceraldehyde‐3‐phosphate dehydrogenase; IFN‐τ, interferon tau; KRT8, keratin 8; PLAC8, placenta‐specific; R, reverse primer; SLC2A1, solute load carrier family 2 member A1; SOD1, superoxide dismutase 1; SOD2, superoxide dismutase 2.

The level of expression of two constitutive genes, Glyceraldehyde‐3‐phosphate dehydrogenase (GAPDH) and β‐Actin (ACTB), was subjected to the GeNorm analysis program. This gene was then used as a reference to normalize the data. The relative expression of each gene was calculated using the ΔΔCt method with efficiency correction by the Pfaffl method (Livak and Schmittgen 2001).

### 2.5 Evaluation of Blastomeres Number and Detection of Apoptotic Cells

The number of apoptotic cells (NAC) in the embryos was determined by the terminal deoxynucleotidyl transferase (TdT)‐mediated dUTP nick end labeling (TUNEL) assay using the one‐step TUNEL In Situ Apoptosis Kit (Elabscience, Mannheim, GER), according to the manufacturer's instructions. The number of blastomeres was measured using a Hoechst 33342 fluorochrome. To avoid bias during assessment, 20 expanded blastocysts (grade I and II) from each treatment group were evaluated. The embryos (treatments, positive control, and negative control) were washed with 0.1% polyvinylpyrrolidone in phosphate‐buffered saline (PBS/PVP) and then fixed in 3.7% paraformaldehyde diluted in PBS/PVP for 1 h at room temperature. Next, the embryos were incubated in a membrane permeabilizing solution (0.5% Triton X‐100 and sodium citrate in PBS) at 4°C for 1 h. The positive control was incubated in 50 U/mL DNase solution in a humid chamber at 37°C for 1 h, while the other embryos were incubated in PBS/PVP on a heating plate at 37°C. For the TUNEL reaction, the treated and positive control embryos were embedded in the enzyme‐marker solution (1:9), while the negative control was immersed in the marker solution alone, followed by incubation in a humid chamber at 37°C for 1 h in the dark. Next, embryos from all treatment groups were incubated in Hoechst 33342 solution (Invitrogen Co., Carlsbad, CA, USA) for 10 min in the dark. Embryos were deposited on slides under coverslips for analysis under an epifluorescence microscope (Axiophot, Zeiss, German). Between each step of the preparation, the embryos were washed three times: once in PBS‐PVP and twice in the final drop. The nuclei of positive TUNEL cells (fragmented DNA) were visualized at 450 nm (green), and all nuclei were visualized at 365 nm (blue). Quantification was performed using the ImageJ software (NIH, Bethesda, MD, USA). For each embryo, the total number of nuclei stained with Hoechst (total nuclei) and the number of nuclei positive for apoptosis (TUNEL signal) were counted. The apoptosis index was calculated as the ratio between TUNEL‐positive nuclei and the total number of nuclei and was expressed as a percentage.

### 2.6 Statistical Analysis

Twenty replicates of embryo production were performed for each oxygen tension system, with and without melatonin. Continuous variables (mitochondrial activity, lipid metabolism, and gene expression) were compared by ANOVA followed by Tukey's test, with a significance level of 5% (*p* ≤ 0.05). Binomial variables (cleavage and blastocyst rates) were analyzed by the chi‐square test 5% (*p* ≤ 0.05). Data are presented as standard error of mean (m ± SEM). Analyzes were performed using the GraphPad Prism 10 software.

## 3 Results

Melatonin supplementation in the embryo culture medium under low oxygen tension (LO‐MEL+) resulted in a higher cleavage rate compared with the other treatment groups (LO‐MEL − , HI‐MEL+, and HI‐MEL − ). However, no difference in blastocyst rate was observed between LO‐MEL+ and LO‐MEL− (Table 2). In contrast, embryos cultured under high oxygen tension HI‐MEL+ exhibited a higher blastocyst rate than their respective HI‐MEL− (39.80 ± 2.14% vs. 34.04 ± 1.72%, respectively; *p* < 0.05; Table 2), and also compared to the treatments where embryos were cultured under low O_2_ tension (31.67 ± 2.52% vs. 31.39 ± 2.10%, with and without melatonin, respectively).

**Table 2:** Effect of melatonin supplementation on cleavage and blastocyst rates under high and low O_2_ concentrations (m ± SEM).

Treatments	Oocytes	Cleavage rate (%)	Blastocyte rate D7 (%)
High O^2^ tension with melatonin	589	78.50 ± 3.00 (461/589)^b^	39.80 ± 2.14 (233/589)^a^
High O^2^ tension without melatonin	584	76.93 ± 2.43 (447/584)^b^	34.04 ± 1.72 (198/584)^b^
Low O^2^ tension with melatonin	533	82.32 ± 2.16 (437/533)^a^	31.67 ± 2.52 (164/533)^b^
Low O^2^ tension without melatonin	554	76.33 ± 1,83 (417/554)^b^	31.39 ± 2.10 (174/554)^b^

*Note*: ^a,b^ Different superscripts in the same column indicate statistical difference. (*p* ≤ 0.05).

**Figure 1:**
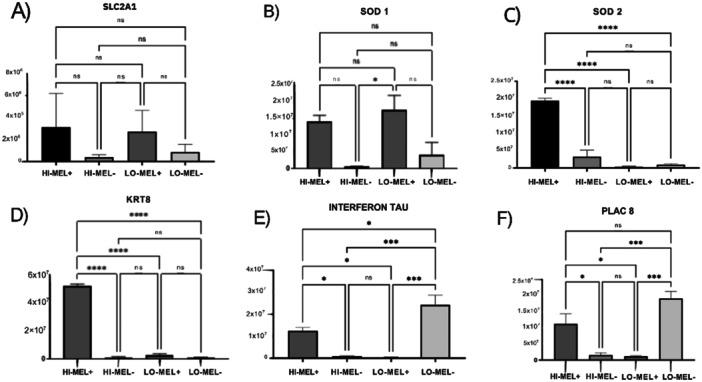
Relative mRNA quantification of the SLC2A1 (A), SOD1 (B), SOD2 (C), KRT8 (D), Interferon Tau (E), and PLAC8 (F) genes in bovine embryos treated LO‐MEL + LO‐MEL− and HI‐MEL + HI‐MEL‐. Quantification was performed by RT‐qPCR, and values were normalized by reference genes. Data are presented as mean ± standard error of the mean (SEM). Asterisks (*, ***, and ****) indicate significant differences between treatments. The more asterisks, the greater the significance (*p* ≤ 0.05); ns, not significant.

The analysis of gene expression in the embryos revealed that there was no difference in the expression of the SLCA1 gene in any of the treatments (*p* > 0.05; Figure 1A).

It was also observed that under high O_2_ tension, the use of melatonin in embryo culture led to higher expression of the SOD2 (*p* < 0.0001; Figure 1C), KRT8 (*p* < 0.0001; Figure 1D), IFN‐τ (*p* = 0.0344; Figure 1E) and PLAC8 (*p* = 0.0307; Figure 1F) genes compared to the treatment without melatonin under high O_2_ tension. There was also higher expression of the SOD2 (*p* < 0.0001; Figure 1C), KRT8 (*p* < 0.0001; Figure 1D), IFN‐τ *p* = 0.0283; Figure 1E), and PLAC8 *p* = 0.0253; Figure 1F) genes in embryos from the melatonin treatment under high O_2_ tension compared to the melatonin treatment under low O_2_ tension.

Melatonin treatment in the embryo culture medium under the low O_2_ tension system did not alter the expression of the evaluated genes compared to the control without melatonin (*p* > 0.05). Only the SOD1 gene presented increased expression in the melatonin treatment under low O_2_ tension compared to the treatment without melatonin under high O_2_ tension (*p* = 0.0190; Figure 1B).

Conversely, it was observed that embryos cultured in the absence of melatonin under low O_2_ tension showed higher expression of the IFN‐τ *p* = 0.0005; Figure 1E) and PLAC8 (*p* = 0.0008; Figure 1F) genes. In turn, interferon gene expression in embryos cultured under low O_2_ tension without melatonin was also higher than all other treatments evaluated under high O_2_ tension.

**Figure 2:**
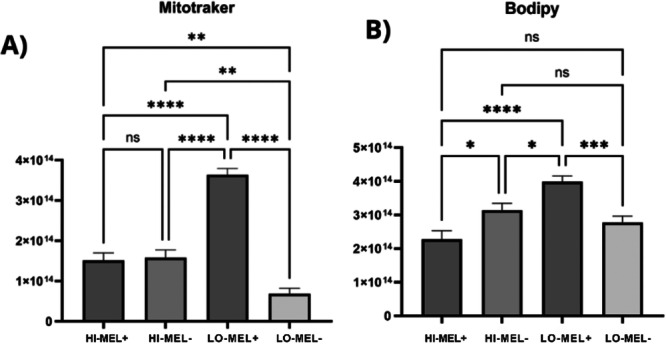
Effect of melatonin on mitochondrial activity (A) and (B) lipid accumulation in bovine embryos under different oxygen tension conditions. The Y‐axis unit represents the fluorescence intensity of the Mitotracker (for mitochondria) and Bodipy (for lipids) markers. Asterisks (*, ***, and ****) indicate significant differences between treatments. The more asterisks, the greater the significance (*p* ≤ 0.05); ns, not significant.

The assessment of mitochondrial function showed that melatonin supplementation in the culture medium increased mitochondrial activity in embryos cultured under low O_2_ tension (*p* = 0.0001; Figures 2A; and 3C,D) compared to all other treatments.

**Figure 3:**
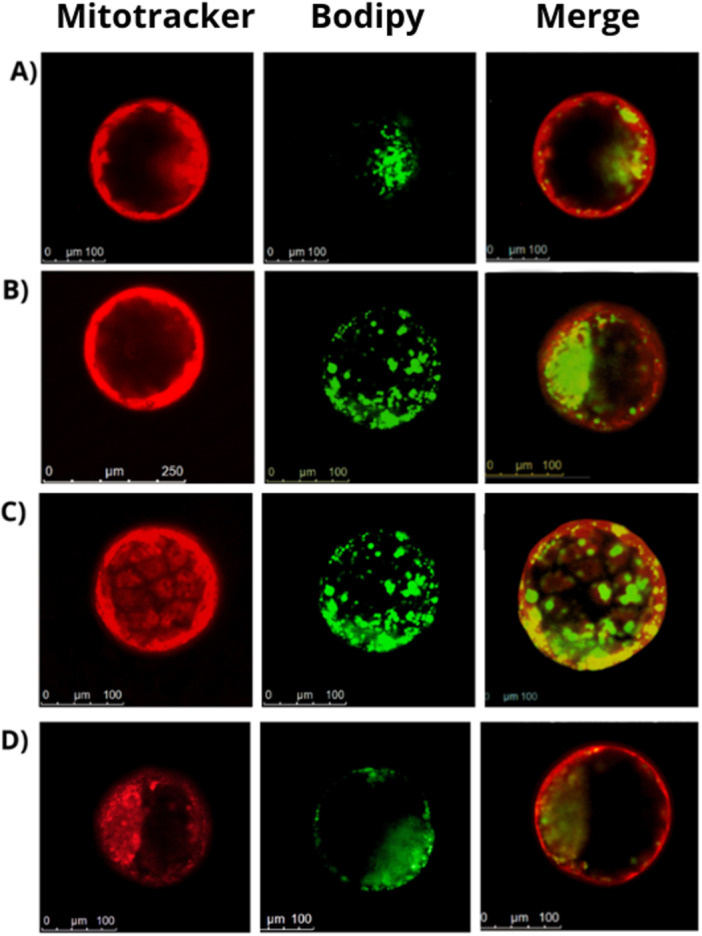
Mitochondria and lipids markers in bovine blastocysts produced *in vitro* under different oxygen conditions and melatonin supplementation. The red marker (Mitotracker) indicates mitochondrial intensity, while the green staining (Bodipy) represents lipid labeling in blastocysts. Embryo culture with melatonin (A) and without melatonin (B) under high O_2_ tension; Embryo culture with melatonin (C) and without melatonin (D) under low O_2_ tension. Scale bar 100 µm.

Under high O_2_ tension there was no difference in mitochondrial function in embryos cultured with and without melatonin (*p* > 0.05; Figures 2B; and 3A,B).

Furthermore, the presence of melatonin significantly reduced lipid accumulation in embryos cultured under high O_2_ tension (*p* = 0.0174; Figures 2B; and 3A,B), but it had the opposite effect on embryos cultured under low O_2_ tension compared to the treatment without melatonin (*p* = 0.0002; Figures 2B; and 3B,D).

The analysis of the total number of blastomeres and apoptotic blastomeres among the different experimental treatments revealed statistically significant differences (*p* < 0.05; Table 3).

**Table 3:** Total number of blastomeres and percentage of apoptosis in bovine blastocysts cultured under different oxygen tension conditions, with or without melatonin supplementation (m ± EP).

Treatments	Total number of blastomeres	Number of apoptotic blastomeres (m ± SEM)	Apoptosis percentage
High O^2^ tension with melatonin	190.00 ± 1.47^a^	4.50 ± 0.24	2.36%^a^
High O^2^ tension without melatonin	144.40 ± 2.58^c^	10.65 ± 0.51	7.37%^b^
Low O^2^ tension with melatonin	178.40 ± 2.82^b^	6.20 ± 0.48	3.47%^c^
Low O^2^ tension without melatonin	179.45 ± 2.90^b^	4.95 ± 0.36	2.75%^a,c^

*Note*: ^a,b,c^ Different superscripts in the same column indicate statistical difference. (*p* ≤ 0.05).

Embryos cultured under high O₂ tension with melatonin supplementation (HI‐MEL+) showed the highest total number of blastomeres (190.00 ± 1.47) and the lowest apoptosis percentage (2.36%), indicating a positive effect of melatonin on embryo quality in this oxidative environment. Conversely, embryos cultured under high O₂ tension without melatonin (HI‐MEL − ) had the lowest total number of blastomeres (144.40 ± 2.58) and the highest number of apoptotic cells (10.65 ± 0.51), resulting in the highest observed apoptosis rate (7.37%), the detrimental effects of high O₂ tension in the absence of antioxidant supplementation.

In embryos cultured under O_2_ tension, the total number of blastomeres was similar between the treatments with and without melatonin (178.40 ± 2.82 e and 179.45 ± 2.90, respectively). However, the treatment without melatonin showed a lower apoptosis rate than the treatment with melatonin (*p* < 0.05). This apoptosis rate in the blastomeres was similar to that found in embryos cultured with melatonin under high O_2_ tension (*p* > 0.05).

In summary, Supporting Information Figure S1 demonstrates, in an integrative heat map, the coordinated effects of melatonin on developmental outcomes, metabolism, apoptosis, and gene expression in bovine embryos cultured under different oxygen tensions.

## 4 Discussion

The results of this study demonstrated that under conditions of low O_2_ tension, melatonin did not influence the blastocyst rate, although it did promote an increase in mitochondrial activity in embryos. Conversely, supplementation with melatonin in the embryo culture medium under high O_2_ tension promoted a significant increase in the blastocyst rate on Day 7, and in the expression of genes related to oxidative stress. The embryo quality was also enhanced in this last treatment as per the reduced apoptosis rate and cytoplasmic lipid accumulation. These findings reinforce the hypothesis that melatonin exerts a protective effect against damage induced by reactive oxygen species (ROS), which are more prevalent in environments with greater O_2_ availability, and can also positively modulate embryo quality, preparing the embryo for cryopreservation or uterine implantation.

The higher blastocyst rate observed in this study in the high O_2_ tension with melatonin treatment (Table 3), compared to the other treatments, may be associated with the reduction of oxidative stress, control of apoptosis, and regulation of genes related to cell survival. Such effects may contribute to greater efficiency in embryo production and, potentially, to a higher number of pregnancies. Our results corroborate the findings of Marques et al. (2020), who also observed an increase in blastocyst production with melatonin supplementation in the culture medium. Similarly, Gutiérrez‐Añez et al. (2021) reported a beneficial effect of a different concentration of this hormone on oocyte maturation, indicating that the positive action of melatonin may encompass different phases of the initial development.

Similarly, previous studies have reported beneficial effects of melatonin supplementation during *in vitro* embryo production in bovine and other species. For instance, Papis et al. (2007) demonstrated improvements in bovine embryo development following melatonin supplementation; however, differences in experimental design, such as oxygen tension, melatonin concentration, and evaluated endpoints, limit direct comparisons with the present study. Notably, our experimental model allowed the investigation of mitochondrial activity, lipid accumulation, apoptosis, and the expression of genes related to oxidative stress and embryo competence, providing mechanistic insights into how melatonin exerts protective effects, particularly under high O₂ tension. In addition, studies in murine and other animal models have consistently shown that melatonin improves embryo quality by reducing oxidative stress and apoptosis, suggesting that its protective role during early embryogenesis is conserved across species (Wang et al. 2013; Gao et al. 2012; Zhao et al. 2017).

In this study, it was observed that melatonin exerted modulatory effects on the expression of antioxidant and embryo quality‐related genes, especially under high oxygen tension conditions. Specifically, the expression of the SOD2 gene, whose corresponding enzyme is fundamental in the biotransformation of reactive oxygen species within the mitochondrial matrix (Halliwell and Gutteridge 2015; Wang et al. 2021), was significantly higher in the melatonin treatment under high O_2_ tension. This increase in SOD2 expression is consistent with the literature, which describes its induction as an adaptive response to increased cellular oxidative load, being less pronounced in physiologically stable environments with low O_2_ tension (Ofosu et al. 2023). Therefore, the present finding reinforces melatonin's role as an agent that optimizes the mitochondrial stress response, enhancing the embryo's ability to counteract the deleterious effects of an environment with oxygen in excess.

In contrast to what was observed for SOD2 under high O_2_ tension, the expression of the SOD1 gene remained unchanged regardless of melatonin treatment. The stability in SOD1 expression in a low oxygen tension environment is consistent with previous studies, which indicates that its regulation is less sensitive to exogenous modulators when cellular oxidative stress is low (Fang et al. 2019). In physiological environments, the basal antioxidant machinery, including the constitutive activity of SOD1, is apparently adequate to maintain redox homeostasis, not requiring additional gene induction mediated by melatonin. This reinforces the hypothesis that the role of melatonin in regulating antioxidant genes is context‐dependent.

Additionally, a higher expression of the IFN‐τ, KRT8, PLAC8 genes was observed in embryos cultured under high O_2_ tension with melatonin. IFN‐τ is a fundamental cytokine secreted by the trophectoderm during the blastocyst stage and plays an essential role in maternal recognition of pregnancy. It inhibits the expression of endometrial oxytocin receptors and consequently prevents the pulsatile release of prostaglandin F2α (PGF2α), the main luteolytic agent (Bazer et al. 2017; Forde and Lonergan 2017). However, *in vitro* embryo production frequently results in lower IFN‐τ expression, a phenomenon associated with the high oxidative stress that cells undergo during the process (Aguilera et al. 2023; Takahashi et al. 2003). In this context, our results suggest that melatonin, due to its known antioxidant function, acted by minimizing the deleterious effects of the culture environment. Consequently, medium supplementation favored the expression of IFN‐τ, a key marker of embryonic competence and viability.

The KRT8 gene is an epithelial intermediate filament involved in trophectoderm differentiation and epiblast development (Vijayaraj et al. 2009; Colitti et al. 2007). Curiously, the increased expression we observed in this study contrasts with the data of Marques (2020), in a study which did not detect an effect of melatonin supplementation on KRT8 expression in vitrified embryos. This difference may lie in the experimental model, since the stress induced by high oxygen tension may affect molecular pathways distinct from those impacted by the cryopreservation process. Thus, the increase in KRT8 expression described here indicates that melatonin actively promotes viability and correct cellular differentiation, especially of the epiblast, in embryos under oxidative stress.

The increased expression of the PLAC8 gene observed in the melatonin‐treated group is a relevant indication of improved embryonic quality, given that this gene is fundamental for trophoblast development and implantation success. Our results, in line with the literature (Marques et al. 2020), suggest that melatonin protects the embryo from damage caused by the oxidative stress imposed by high oxygen tension. Similarly, Monteiro et al. (2024) reported a beneficial effect of the hormone at a different concentration on oocyte maturation, reinforcing the positive action of melatonin on different phases of initial development. The PLAC gene expression increase observed in this study reinforces melatonin's role as a positive modulator of molecular pathways essential for embryo viability and implantation competence.

Under low O_2_ tension, embryos in the control group (without melatonin) showed higher expression of IFN‐τ than all other treatments. PLAC8 gene increased expression was also observed, except when melatonin was used in high O_2_ tension. This finding suggests that the low‐tension environment represents a favorable physiological condition for the adequate expression of genes crucial for implantation. This point is corroborated by studies demonstrating the capacity of low O_2_ tension to positively modulate these gene pathways (Colitti et al. 2007; Zeng et al. 2024) without the need for exogenous antioxidants. Therefore, the benefit of melatonin is context‐dependent, and it is more significant as a protective agent under conditions of high oxidative stress.

In addition to its effects during embryo culture, melatonin is physiologically present in the follicular environment and plays a relevant role during oocyte maturation. Several studies (Zhao et al. 2019) have demonstrated that melatonin supplementation during *in vitro* maturation (IVM) improves oocyte quality, mitochondrial function, and subsequent embryo development. Therefore, although the present study focused on melatonin supplementation during embryo culture, the combined use of melatonin during both IVM and embryo culture represents a promising strategy for further optimization of *in vitro* systems. This integrated approach may better mimic the *in vivo* reproductive environment and further enhance embryonic competence.

The evaluation of mitochondrial activity demonstrated that embryos supplemented with melatonin in a low O_2_ tension environment showed the highest metabolic functionality among all groups (Figure 2A). The increase in mitochondrial activity is directly associated with higher ATP production and superior embryonic quality and viability, as described by Lira et al. (2020). The apparent dissociation between gene expression and mitochondrial function is explained by the fact that melatonin possesses multiple mechanisms of action, and even in a low oxidative stress environment, melatonin acts to increase energy production, which would boost embryonic competence. In turn, under low O_2_ tension melatonin supplementation resulted in higher lipid accumulation, suggesting a possible decrease in lipolysis, leading to the controlled storage of energy reserves. Embryos cultured without melatonin under low O_2_ tension, however, showed a reduction in lipid accumulation, similar to what was observed in embryos cultured in the presence of melatonin under high O_2_ tension. This finding demonstrates that the embryo culture system under low O_2_ tension is more physiological and demands fewer compensatory responses to oxidative stress.

Another highlight of melatonin supplementation in embryo culture under high O_2_ tension was the significant reduction observed in the accumulation of intracellular lipids in the embryos (*p* = 0.0174; Figures 2B and 3), compared to the treatments without melatonin under high O_2_ tension and with melatonin under low O_2_ tension. This protective effect is consistent with the powerful antioxidant role of melatonin, which limits the formation of reactive oxygen species (ROS) and subsequent lipid peroxidation, as demonstrated in several models (Lira et al. 2020; Morvaridzadeh et al. 2020). In our study, the lipid reduction in embryos promoted by melatonin under high O_2_ tension can be explained by the decrease in oxidative stress and mitochondrial stabilization, factors that favor efficient energy metabolism and consequently reduce lipid accumulation. Thus, the use of melatonin can be an important strategy for the cryopreservation of *in vitro‐*produced embryos, provoking a reduction in lipid droplets, which are detrimental to the embryos during this process. Conversely, under low O_2_ tension, melatonin supplementation resulted in greater lipid accumulation, suggesting a possible decrease in lipolysis, leading to the controlled storage of energy reserves. In contrast, embryos cultured without melatonin under low O_2_ tension showed reduced lipid accumulation, which was similar to that observed in embryos cultured in the presence of melatonin under high O_2_ tension.

It is important to consider that the culture system used in the present study contained fetal bovine serum (FBS), which, although widely employed due to its capacity to support embryo development, is also associated with alterations in embryo metabolism, lipid accumulation, and long‐term developmental competence. Previous studies using serum‐free or protein‐defined culture systems have demonstrated that melatonin exerts beneficial effects independently of serum supplementation, suggesting that its antioxidant and cytoprotective actions are intrinsic (Gupta et al. 2008; Wang et al. 2013). However, the presence of FBS in the culture medium may partially mask or modulate the magnitude of melatonin's effects. Therefore, future studies evaluating melatonin supplementation under serum‐free conditions may help to further elucidate its isolated contribution to embryo quality and developmental competence.

Additionally, melatonin supplementation under high O_2_ tension also demonstrated a positive effect regarding a higher total number of blastomeres (*p* < 0.05) and, crucially, a drastic reduction in the proportion of apoptotic cells, from 7.37% (melatonin‐free treatment) to 2.36% (melatonin treatment). This anti‐apoptotic effect corroborates the findings of (Su et al. 2015; Remião et al. 2016; Komninou et al. 2016; Hitit et al. 2023), which describe melatonin's capacity to suppress the caspase pathway and the expression of pro‐apoptotic genes (BAX, CASP3), while potentiating the endogenous antioxidant response (SOD2, GPX1). Thus, the preservation of cell mass through the inhibition of apoptosis explains the higher number of blastomeres, a clear indication of higher embryonic viability.

## 5 Conclusion

The presence of melatonin in an embryo culture system with high oxygen tension provided significant benefits to embryo quality and viability, acting as an antioxidant agent, gene modulator, and apoptosis reducer. These findings reinforce the potential of melatonin as an adjuvant in embryo culture, especially in environments where exposure to atmospheric oxygen is unavoidable. Conversely, melatonin supplementation in an embryo culture system with low O_2_ concentration demonstrated a limited impact on embryo development and quality. Since this system is closer to the physiological environment and less challenging for ROS production, the addition of an antioxidant molecule seems unnecessary, even considering melatonin's additional properties.

## Author Contributions

**Isabella Rodrigues dos Santos Oliveira:** investigation, methodology, writing – review and editing, conceptualization. **Carlos Frederico Martins:** conceptualization, investigation, project administration, writing – review and editing. **Fabiana Lima Rodrigues:** investigation, methodology. **Victor Carlos Mello:** investigation, methodology. **Maria Tereza de Oliveira Rodrigues:** investigation, methodology. **Lucas Costa de Faria:** investigation, methodology. **Hallya Beatriz Sousa Amaral:** investigation, methodology. **Rosângela Vieira de Andrade:** investigation, methodology. **Marcio José Poças Fonseca:** investigation, methodology. **Margot Alves Nunes Dode:** investigation, methodology. **Sônia Nair Báo:** conceptualization, funding acquisition, writing – review and editing, project administration.

## Conflicts of Interest

The authors declare no conflicts of interest.

## Supporting information


**Figure S1:** Integrative heatmap summarizing the coordinated effects of melatonin on developmental, metabolic, apoptotic, and gene expression outcomes in bovine embryos cultured under different oxygen tensions.
