# Supplementary material for: The Impact of Melatonin on Cellular Dynamics and Gene Expression of Bovine Embryos Cultured Under Low and High Oxygen Tension
Source: Mol Reprod Dev. 2026 Apr 6;93(4):e70102. doi: 10.1002/mrd.70102 (PMC13051412; doi:10.1002/mrd.70102)
Supplement: Supplementary file 1 — Figure S1: Integrative heatmap summarizing the coordinated effects of melatonin on developmental, metabolic, apoptotic, and gene expression outcomes in bovine embryos cultured under different oxygen tensions. [file MRD-93-e70102-s001.docx]

*Supplementary Material*

The Impact of melatonin on cellular dynamics and gene expression of bovine embryos cultured under low and high oxygen tension.

Isabella Rodrigues dos Santos Oliveira ^(1,2)^; Carlos Frederico Martins^(2)^ ; Fabiana Lima Rodrigues ^(2,3)^;Victor Carlos Mello ^(1);^ ; Maria Tereza de Oliveira Rodrigues ^(1)^; Lucas Costa de Faria ^(2, 6);^; Hallya Beatriz Sousa Amaral ^(5)^ ;Rosângela Vieira de Andrade ^(5);^ Marcio José Poças Fonseca ^(1)^; Margot Alves Nunes Dode ^(4)^; Sônia Nair Báo^(1)^

¹ Departamento Biologia Celular, Universidade de Brasília, Brasília-DF, Brasil.

^2^ Embrapa Cerrados, Brasília-DF, Brasil.

^3^ Icesp, Brasília- DF, Brasil.

^4^ Embrapa Recursos Genéticos e Biotecnologia, Brasília- DF, Brasil.

^5^ Programa de Pós-graduação em Ciências Genômicas e Biotecnologia. Universidade Católica de Brasília, Brasília- DF, Brasil.

^6^ Departamento Saúde Animal, Universidade de Brasília, Brasília-DF, Brasil.

*Corresponding Author – snbao@unb.br


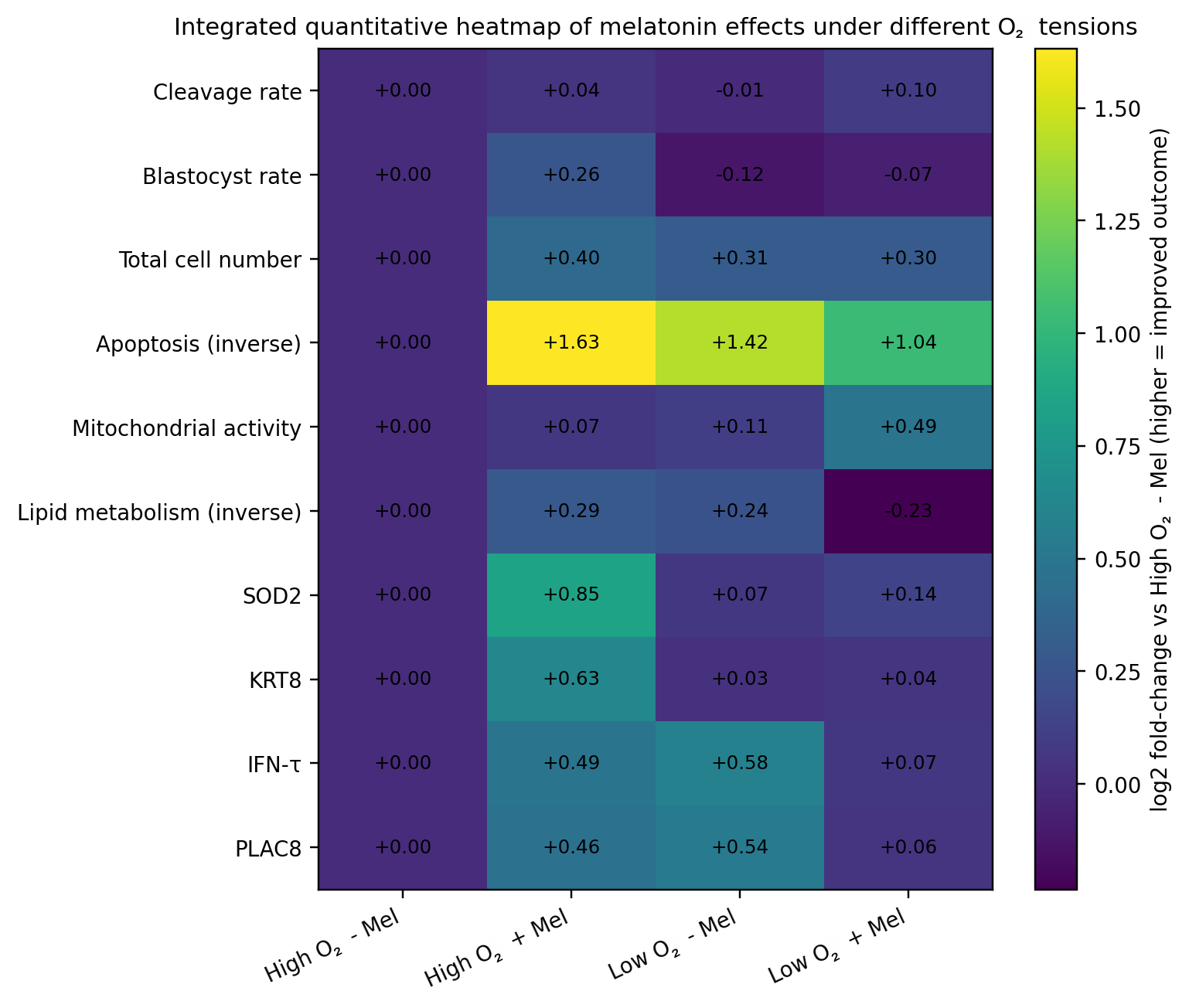


**Figure S1** - Integrative heatmap summarizing the coordinated effects of melatonin on developmental, metabolic, apoptotic, and gene expression outcomes in bovine embryos cultured under different oxygen tensions.

Embryos were cultured under high (20% O₂) or low (5% O₂) oxygen conditions in the presence or absence of melatonin. Quantitative outcomes, including cleavage rate, blastocyst rate, total cell number, apoptosis, mitochondrial activity, lipid metabolism, and the expression of genes associated with oxidative stress and embryo competence (SOD2, KRT8, IFN-τ, and PLAC8), were integrated into a single visualization framework. Values are expressed as log₂ fold-change relative to the high oxygen control group without melatonin, which was set as the baseline reference (zero). For parameters associated with detrimental effects, such as apoptosis and lipid accumulation, values were inverted to reflect biological improvement.

Color intensity represents the magnitude and direction of the response, allowing direct comparison across endpoints with different measurement scales. This integrative analysis highlights a coordinated improvement in embryonic competence under high oxygen tension with melatonin supplementation, characterized by increased blastocyst formation, higher cell numbers, reduced apoptosis, decreased lipid accumulation, and upregulation of antioxidant and implantation-related genes. In contrast, embryos cultured under low oxygen tension exhibited a more limited and context-dependent response, with predominant modulation of mitochondrial activity and minimal changes in developmental outcomes.

Together, this visualization supports the hypothesis that melatonin exerts a context-dependent cytoprotective effect, acting primarily as an antioxidant and metabolic modulator under oxidative stress conditions, while its impact is reduced in physiologically low oxygen environments.
